# On the use of a continuous thrust to find bounded planar trajectories at given altitudes in Low Earth Orbits

**DOI:** 10.1038/s41598-020-65594-w

**Published:** 2020-05-26

**Authors:** Allan Kardec de Almeida, Jhonathan Orlando Murcia Piñeros, Antonio Fernando Bertachini de Almeida Prado

**Affiliations:** 1 INPE: Instituto Nacional de Pesquisas Espaciais, São José dos Campos, SP Brazil; 2 Aerospace Engineering, Texas A&M University, College Station, TX USA

**Keywords:** Space physics, Astronomy and astrophysics

## Abstract

In this work, it is shown how a spacecraft equipped with a thrust and subjected to a drag force can be bounded at specific altitudes as function of the parameters of the thrust. It is used nonlinear dynamics tools to find attractors, which bound the motion of the spacecraft. For a specific set of parameters of the thrust, the spacecraft is bounded to a given altitude. Several forms for the thrusts are proposed in order to bound the altitude of the spacecraft. The influence of several forms of perturbations in the altitude of the spacecraft is also investigated in this work, like the solar radiation pressure, gravity of the Moon and oblateness of the Earth. Finally, nonlinear dynamics tools are also used to investigate transfers among the bounded orbits in different altitudes.

## Introduction

The number of satellites in Low Earth Orbits (LEOs) is increasing due to the development of Small Sats missions. Over 1,000 smallsats were launched from 2012 to 2017, according to [Smallsats by the Numbers 2018, Bryce Space and Technology]. Satellites in the Low Earth Orbits (LEOs) are highly perturbed by drag^1^, among other forces. The atmospheric drag can be used in maneuvers around the Earth and planets like Mars or Venus^2–4^. In general, some kind of thrust is necessary to counteract the losses of altitude and energy due to the atmospheric drag. Orbit maintenance may be performed by using continuous or impulsive maneuvers to return the satellite to near its initial orbit. In the present work, several forms of continuous thrusts are defined and studied with the objective to select the ones that give trajectories that are bounded^5^ at specific altitude regions. In that sense, the present paper proposes solutions to keep a spacecraft in a given orbit by applying a thrust that continuously compensate the perturbations received from the environment. Depending on the perturbation, the spacecraft is bounded to a circular orbit or to an elliptic, but periodic orbit in a proper reference frame. Regarding the applied control, several forms are studied. The most complete one has a component proportional to the square of the velocity of the spacecraft, to compensate the effect of drag; another component proportional to the altitude of the spacecraft, to be able to adjust the orbit to fit a desired altitude; and one more component proportional to the ratio of the velocity and the altitude of the spacecraft, to give more controllability to the spacecraft by adding one more degree of freedom to the system. A similar concept is applied in science missions, which goal is to measure the gravitational field, the so called drag-free missions, which are characterized by the use of control techniques to reduce the disturbances affecting the spacecraft, mainly drag. Some examples of these missions are available in references^6–8^.

Although dissipative forces like drag require consumption of fuel to be balanced, in terms of nonlinear dynamics, it enables the existence of attractors^9,10^. In this work, the drag is used to help the mission, because we take advantage of the limited boundaries of the attractors to keep the trajectories in desired specific altitude regions. The main objective of this work is to use attractors to bound trajectories in strategic regions of the atmosphere. In order to better study these attractors, a Poincaré section^11^ will be used to reduce the dimension of the system and to locate it in the phase-space domain.

Besides drag, other perturbative forces also influences the motion at LEOs, like the ones that come from the solar radiation pressure, the oblateness of the Earth and the gravitational attraction of the Moon. Thus, additionally, the influence of these perturbations over the attractors - their displacement in the phase-space - will also be investigated.

Finally, we introduce a method to interpret the successfulness of a transfer for a spacecraft among attractors with different altitudes by the use of their basins of attraction in the phase-space of the Poincaré section. The idea of this technique is to combine a transfer orbit with the requirements to keep the spacecraft in a given attractor. Changing the parameters of the control remove the spacecraft from its attractor and, under some conditions, send it to another attractor. The present study maps these conditions, showing the destiny of the spacecraft after a change in those parameters. It may go to another attractor, to reentry the atmosphere of the Earth or to leave the orbit of the Earth.

The system used in this work is based in the Tsien problem^12^, which represents a satellite orbiting the Earth subjected to a thrust. A dissipative force will be introduced in the system, which represents the drag force that a satellite is subjected due to the atmosphere density. This dissipative force will be used to send the system to specific attractors in desired altitudes of the atmosphere. In a consecutive analysis, perturbative forces will be included, which represent the effects due to the solar radiation pressure over the satellite, the *J*_2_ potential term due to the oblateness of the Earth and the perturbation due to the gravitational interaction with the Moon. The effects of these additional perturbations over the attractors are investigated through their dependence on the values of the parameters of the perturbed system.

The problem of stability of the attractors is also considered. A study is made to show the linear stability of the attractors, helping to decide if it is a good location to place a spacecraft.

Note that in the present research, the orbital maintenance is not applied in the traditional way, i.e., to return the satellite to the operational orbit after some time to compensate the effects of perturbations such that the fuel consumption is minimized, with multiple impulses periodically or low thrust applied in the most efficient regions. The problem under study is how to apply a continuous thrust to keep the spacecraft in a given altitude, not allowing oscillations in the orbit that may minimize fuel consumption. Such situation may be required in a mission, or in part of mission, where observations are required to be made from a given fixed range of altitude. In this case, it is applied a continuum force to transport the satellite into an attractor that lies in the desired altitude, maintaining it in this region under the continuum influence of several types of perturbations. Then, the continuous thrust is necessary to keep the satellite in this region. To do that, one of the best technological methods available is the application of low thrust with electrical propulsion systems. Of course it is possible to find other strategies that may consume less fuel, but they do not keep the spacecraft in a fixed altitude, which may be required sometimes.

In Sec. 2, the models used to formulate the problem are shown. The results for the satellite subjected to drag, perturbations and different forms of thrusts are showed in Sec. 3.

## Formulation of the problem

The Tsien problem^12^ consists in a set of two bodies: a main central body and a second one with negligible mass (the spacecraft) moving around the center of mass of the central body and subjected to a thrust. Additionally to the thrust, a dissipative drag force ($\overrightarrow{D}$) due to the atmospheric interaction with the spacecraft is always considered in the problem. Other perturbations are also taken into consideration for some cases. Using an inertial frame of reference centered in the Earth, the equation of motion of such a spacecraft is

1 $$\overrightarrow{\ddot{r}}=-\,\mu \frac{\overrightarrow{r}}{{r}^{3}}-\beta v\overrightarrow{v}+\tau \overrightarrow{\theta }+\overrightarrow{P},$$

where $\overrightarrow{r}$ is the position vector of the spacecraft with magnitude *r*. The velocity vector is defined by $\overrightarrow{v}=d\overrightarrow{r}/dt$, with magnitude *v*. *τ* is the magnitude of the specific thrust to be studied; $\overrightarrow{\theta }$ is a unit vector perpendicular to $\overrightarrow{r}$, defined according to the traditional polar coordinates; *μ* is the gravitational parameter of the main body and $\beta v\overrightarrow{v}$ is the term due to the drag, which is in the opposite direction to the velocity $\overrightarrow{v}$ of the spacecraft; *β* = *ρB*, where *ρ* is the atmosphere density and *B* is the Ballistic Coefficient (*B* = *AC*_*D*_/2 *m*), where *C*_*D*_ is the drag coefficient, *A* the transversal area and *m* is its mass^13^. In this work, *ρ* is assumed to be constant for low oscillations of the altitude, around 600 *km*. The density is evaluated using the model NRLMSISE-00^14^ for 600 *km*. The vector $\overrightarrow{P}$ represents the perturbations due to Solar Radiation Pressure, oblateness of the central body (Earth) or third body influence (Moon). These perturbations are better described next.

The Oblateness of the Earth modelled with the term of the acceleration due to *J*_2_ - the second term of the gravity potential of the Earth^1^ - is considered in this work. In this way, the gravitational potential of the Earth is given by

2 $$U=-\,\frac{\mu }{r}-\frac{3{J}_{2}\mu }{2r}\frac{{r}_{e}^{2}}{{r}^{2}}\left({\sin }^{2}\phi -\frac{1}{3}\right),$$

where *ϕ* is the latitude and *r*_*e*_ the mean equatorial radius. The planar orbits analyzed here are restricted to the equatorial and polar planes (*ϕ* = 0 and *ϕ* = ±*π*/2, respectively), which are two of the most important cases, among all the values of *ϕ*.

The acceleration due to a thrust given by a planar solar sail attached to the satellite coming from the solar radiation pressure is given by^15^

3 $${\overrightarrow{P}}_{SRP}=\delta \frac{A}{m}\frac{k{\cos }^{2}(\gamma )}{{\Vert {\overrightarrow{r}}_{s}\Vert }^{2}}\overrightarrow{n},$$

where *δ* is a positive non-dimensional parameter less than 1 (*δ* = 1 means a perfect reflection); *k* is a parameter that depends on the luminosity of the body (*k* = 2*p*_*e*_*R*^2^ in the case where the Sun is the source, where *p*_*e*_ = 4.56 × 10^−6^ *kg*/(*ms*^2^) holds for the solar radiation pressure at a Sun-Earth distance from the Sun); *A* is the area of the flat solar sail; *m* is the mass of the satellite; ${\overrightarrow{r}}_{s}$ is the vector that locates the sail from the source of photons; $\overrightarrow{n}$ is the vector normal to the solar sail and *γ* is the angle between ${\overrightarrow{r}}_{s}$ and $\overrightarrow{n}$, which is given according to $\cos \,\gamma =({\overrightarrow{r}}_{s}\cdot \overrightarrow{n}/{r}_{s})$. In our evaluations, $\overrightarrow{n}$ is set in the direction of *r*_*s*_, the ratio area-to-mass is 5.562 *m*^2^/*kg* and *δ* = 0.0026. The parameter *δ* is used to represent many forms of practical applications, such as a satellite which reflexivity of its panel can be changed^16,17^, as well as a satellite which effective area is adjustable^18,19^. The source of photons (the Sun) is assumed to rotate around the Earth in a circular orbit. However, the vector ${\overrightarrow{r}}_{s}$ is a function of time, so the equations of motion are explicit functions of time, which means that the dimension of the phase-space will be increased by one. In order to avoid this issue, a rotating frame of reference that rotates around the Earth with the same angular velocity of the Sun is used. This system does not depend explicitly on time anymore and the dimension of the phase-space is unchanged taking into account the perturbation. Additionally, the solar rays are assumed to be parallel in the vicinity of the Earth, thus a shadow function is included, which means that the solar pressure effects are turned off while the spacecraft passes behind the Earth with respect to the Sun.

The gravity field of the Moon is also included in the system. Our model assumes that the Moon is rotating around the Earth in a circular orbit. Once again, the gravitational term due to the Moon would be explicitly time dependent, thus the system is written in a rotating frame of reference that rotates with the same angular velocity of the Moon. In this new frame of reference, the equations of motion are not explicitly dependent on time, so the dimension of the phase-space is unchanged in comparison with the unperturbed case, when the perturbation of the Moon is not applied. The satellite is assumed to be in an orbit co-planar to the Earth-Moon orbital plane.

These perturbations represent the highest forces at LEO^1,20^ and they are taken into consideration individually. The equation of motion is numerically integrated through a Runge Kutta F-7/8^21^ numerical integrator. A Poincaré section is analyzed to get a better visualization of the attractors in the phase-space. The Poincaré section is defined in the right half-plane *y* = 0. In the case studied here, the motion is restricted to a plane, thus the Poincaré section is defined where the trajectory crosses the value *y* = 0 in the positive side of the *x* axis, which value is *y* = 0. It means that the phase-space in this section has three dimensions. Unless stated otherwise, some other considerations are used in this paper, as show next.

- The spacecraft is initially inserted in a Keplerian circular orbit of radius *a*_0_ around the Earth, so its velocity is given by ${v}_{0}=\sqrt{\mu /{r}_{0}}$.
- The altitude *alt* is defined as *r* − *r*_*e*_, where *r*_*e*_ = 6378.136 *km* is the mean equatorial radius of the Earth.
- To show the parameters in a more suitable form in the results, a unit of length (*uL*) is defined as *uL* = 6978.136 *km* and the unit of time is such that the period of a keplerian orbit with semi-major axis *uL* is 2*π*, so $uT=\sqrt{{(uL)}^{3}/\mu }$.
- In those equations, *μ* is the gravitational parameter of the Earth. To have an idea of the parameter *β*, for the International Space Station it is ${\beta }_{ISS}=1.53055116\times {10}^{-14}\,{m}^{-1}$ for altitudes around 600 *km*, which corresponds to a ratio area-to-mass 0.005562 *m*^2^/*kg*. To keep the transient in a reasonable time scale, in the order of a few years, the parameter *β* is fixed in *β* = *β*_*Iss*_ × 10^3^, which corresponds to a spacecraft with the same drag coefficient *C*_*D*_ of the ISS, but an area-to-mass ratio 5.562 *m*^2^/*kg*. The ISS is used as a reference, because it is the largest artificial satellite in LEO. Due to its area-to-mass ratio, it has a rapid decay and, to maintain the ISS in orbit, impulsive maneuvers are made periodically.

Note that basics properties of nonlinear dynamics tools are used in this paper to search for orbits at LEOs, like Poincaré sections, attractors and their basin of attraction for the dynamical system considered. Attempts to rigorous definition of such tools are extensively available in the literature^9–11^.

## Results

For all kinds of thrusts, the case where there are no perturbations acting in the system is initially investigated, i.e. $\overrightarrow{P}=\overrightarrow{0}$ in Eq. 1, where $\overrightarrow{0}$ is the null vector. The system will be then subjected to several types of perturbations: the solar radiation pressure^15^, the *J*_2_ term of the gravitational potential of the Earth^1^ for polar and equatorial orbits and the gravitational interaction with the Moon^20^. This process will be done for several forms of thrust. Firstly, two forms for the thrust will be independently investigated in subsections 3.1 and 3.2. Then, a new form that includes all the terms added will be proposed and investigated in subsection 3.3.

### Thrust proportional to the radius

The first form of the thrust to be investigated is the case where the magnitude of the thrust is proportional to the radius. In this case, *τ* is given by

4 $$\tau ={\alpha }_{1}({r}_{0}-r)+{\alpha }_{0}\beta {v}_{0}^{2},$$

where *α*_1_ and *α*_0_ are parameters of the magnitude of the thrust and *r*_0_ is a parameter that should be chosen around the desired altitude of the attractor. The second term is a simple constant and its form is selected to approximately compensate the magnitude of the drag in the case where the parameter *α*_0_ equals a unity, since the magnitude of the initial velocity *v*_0_ is near the magnitude of the velocity of the spacecraft in the attractor. The first term of the right side of Eq. (4) is a linear proportional control where *α*_1_ is the proportional gain^22^. Note that it can be seen as a spring-mass system, having a force that obligates the spacecraft to oscillate around *r*_0_. In the case where the spacecraft is falling to the Earth, i.e. *r* < *r*_0_, the force is approximately in the same direction of the velocity, hence its magnitude tends to increase and the spacecraft tends to ascend. On the other side, in the case where the spacecraft is escaping from the Earth, i.e. *r* > *r*_0_, the force is approximately in the opposite direction of the velocity, hence the spacecraft tends to descend. Thus, due to the existence of the drag, the system shall work like a damped oscillator.

**Figure 1:**
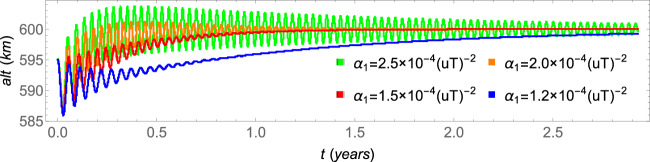
The evolutions to the same attractor for different values of the parameter *α*_1_. The overdumped pattern cases are shown in blue and red, while the underdumped pattern cases are shown in orange and green. The other parameters are *α*_0_ = 1, *r*_0_ = *r*_*e*_ + 600 *km* and *a*_0_ = *r*_0_ − 5 *km*.

The parameters *α*_0_ and *r*_0_ are set to *α*_0_ = 1 and *r*_0_ = *r*_*e*_ + 600 *km* (or *r*_0_ = 1*uL*), which corresponds to an altitude equals to 600 *km*. The altitudes as functions of time are shown in Fig. 1, for different values of the parameter *α*_1_.

**Figure 2:**
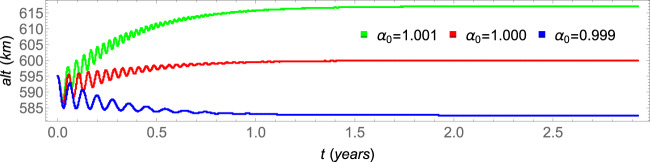
The final altitudes for different values of the parameter *α*_0_. The other parameters are *α*_1_ = 1.5 × 10^−4^(*uT*^−2^), *r*_0_ = *r*_*e*_ + 600 *km* and *a*_0_ = *r*_0_ − 5 *km*.

The spacecraft evolves to the attractors, which coordinates in the phase-space of the Poincaré section are shown in Table 1 for several values of the parameter *α*_1_. Note that the coordinates of all the attractors are almost coincident, but the spacecraft evolves to this attractor in different ways, as shown in Fig. 1. The patterns are analogous to the solutions of the dumped harmonic oscillator: the overdumped pattern cases are shown in blue and red for the parameters *α*_1_ = 1.2 × 10^−4^(*uT*^−2^) and *α*_1_ = 1.5 × 10^−4^(*uT*^−2^), respectively, and the underdumped patterns cases are shown in orange and green, for the parameters *α*_1_ = 2.0 × 10^−4^(*uT*^−2^) and *α*_1_ = 2.5 × 10^−4^(*uT*^−2^), respectively. The time to reach the attractor is lower for values of the parameter closer to *α*_1_ ≈ 1.8 × 10^−4^(*uT*^−2^). For values of the parameter *α*_1_ < 1.1 × 10^−4^(*uT*^−2^), the first term of the right side of Eq. 4 is not strong enough to send the spacecraft to the attractor and, as a consequence, the spacecraft falls to the Earth. On the other side, for values of the parameter *α*_1_ > 2.7 × 10^−4^(*uT*^−2^), the first term of the right side of Eq. 4 is too strong in comparison with the drag, so that the amplitudes of the oscillations become larger along time and, as a consequence, the spacecraft cannot reach the attractor as well. On the other hand, the effect of the parameter *α*_0_ over the altitude of the attractors is shown in Fig. 2, which shows the altitude of the spacecraft for several values of *α*_0_ in the case where *α*_1_ = 1.5 × 10^−4^(*uT*^−2^).

**Table 1:** Coordinates of the attractors in the phase-space of the Poincaré section.

	values for the parameter *α*_1_ (10^−4^(*uT*)^−2^)			
	*α*_1_ = 1.2	*α*_1_ = 1.5	*α*_1_ = 2.0	*α*_1_ = 2.5
*x*(10^6^ *m*)	6.978135999988396	6.978135999998476	6.978135999995473	6.978135999996812
*y*(*m*)	|*y*| < 10^−9^	|*y*| < 10^−9^	|*y*| < 10^−9^	|*y*| < 10^−9^
$\dot{x}(m/s)$	$|\dot{x}| < {10}^{-8}$	$|\dot{x}| < {10}^{-8}$	$|\dot{x}| < {10}^{-8}$	$|\dot{x}| < {10}^{-8}$
$\dot{y}(m/s)$	7557.86574523	7557.86574523	7557.86574523	7557.86574523

#### Linear stability of the unperturbed system

It was observed that the radii *r* of the orbits where the spacecraft is bounded in the attractor vary less than 10^−5^ during one orbital period, for all the attractors shown in Figs. 1 and 2, for the seven combinations of values for *α*_0_ and *α*_1_ used in the simulations made here. Therefore, we can assume that the orbits are circular for those cases. The frame of reference is changed to the one that rotates with the same angular velocity of the spacecraft around the Earth. Hence, in this frame of reference, which takes into account the Coriollis acceleration and other terms^23^, the spacecraft is in an equilibrium state placed at a fixed point. The accelerations evaluated at this point are of the order of 10^−10^ *m*/*S*^2^ or smaller. The linear stability of this fixed point was evaluated by solving the characteristic equation of the linearized system and it is found that all of them are linearly stable, according to the definition given in^24^.

#### Brief conclusion of the unperturbed system

Those parameters (*α*_0_, *α*_1_ and *r*_0_) can be used to adjust the desired altitude for a specific mission. The second term of the right side of Eq. 4 is used to turn the magnitude of the thrust close to the equilibrium with the magnitude of the force due to drag, and it defines the altitude of the attractor, as can be seen in Fig. 2 by the variation of its parameter *α*_0_, while the first term of Eq. 4 defines how the spacecraft is sent to the attractor. Note, from Table 1, that the parameter *α*_1_ does not change the position of the spacecraft in the phase-space of the Poincaré section. They have almost the same coordinates in this phase-space. On the other side, note from Fig. 1 that this same parameter (*α*_1_) highly influences the transient time, i.e., the time the spacecraft takes to reach the attractor. Besides that, it influences the oscillation during this transient, from an overdumped pattern for low values of the parameter to an underdumped one for higher values of *α*_1_. It is also possible to interpret this thrust by using two engines: a constant one that applies the major thrust correspondent to the second term of the right side of Eq. 4, and a variable one that applies the thrust correspondent to the first term of the right side of Eq. 4.

#### Perturbed system

At this stage of the research, perturbations ($\overrightarrow{P}$) will be taken into consideration for a more complete study of the system. Their influence over the attractors will be investigated.

**Figure 3:**
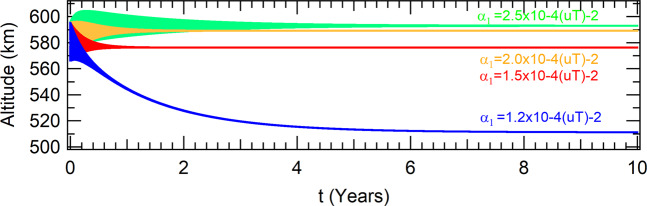
Perturbations due to *J*_2_ in equatorial orbit for a thrust proportional to the radius of the orbit.

The first perturbation added to the system is given by the *J*_2_ term of the Earth gravitational potential, which is due to its oblateness shape. The evolutions of the altitudes in time are shown in Fig. 3, for the same values of the parameters shown in Fig. 1, in the case where the orbit is in the equatorial plane of the Earth. In comparison with the unperturbed problem, the net effect of *J*_2_ is to reduce the altitude of the orbit. This is an expected result, since the *J*_2_ term is equivalent to one increase in the mass of the Earth, for equatorial orbits, in comparison with a spherical Earth. Although the attractor is moved to lower altitudes, the transient time to reach is approximately the same. The values of *α*_1_ between *α*_1_ = 1.5 × 10^−4^(*uT*^−2^) and *α*_1_ = 2.0 × 10^−4^(*uT*^−2^) are the ones in which the spacecraft evolves to the attractor in shorter times. The radius of the orbit changes less than 10^−5^ *m* during the period of an orbit, so the spacecraft is found in a circular orbit which coordinates in the phase-space of the Poincaré section are shown in Table 2.

**Table 2:** Coordinates of the attractors in the phase-space of the Poincaré section using *J*_2_ in equatorial orbits for a thrust proportional to the radius.

	values for the parameter *α*_1_ (10^−4^(*uT*)^−2^)			
	*α*_1_ = 1.2	*α*_1_ = 1.5	*α*_1_ = 2.0	*α*_1_ = 2.5
*x*(*m*)	6889219.09688167	6954284.50657470	6967216.13411273	6971048.23011839
*y*(*m*)	|*y*| < 10^−9^	|*y*| < 10^−9^	|*y*| < 10^−9^	|*y*| < 10^−9^
$\dot{x}(m/s)$	$|\dot{x}| < {10}^{-8}$	$|\dot{x}| < {10}^{-8}$	$|\dot{x}| < {10}^{-8}$	$|\dot{x}| < {10}^{-8}$
$\dot{y}(m/s)$	7611.77483836	7575.98452101	7568.93140867	7566.84508999

**Figure 4:**
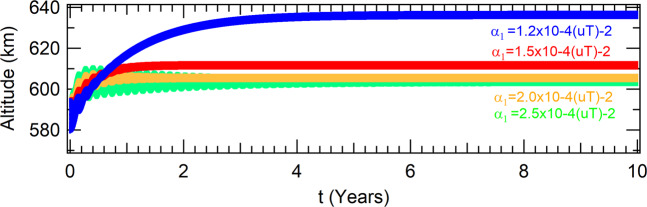
Effects of the perturbation due to *J*_2_ in polar orbit for a thrust proportional to the radius of the orbit in the altitudes of the spacecraft.

In the case of polar orbits, the altitudes of the attractors have an opposite behavior in comparison with the case where the orbit is equatorial. Their altitudes are shifted to higher values. The physical explanation is easy. The *J*_2_ term concentrates more mass around the equator of the Earth, so polar orbits fells a smaller mass acting in their dynamics. They increase as the parameter decreases, as shown in Fig. 4. Although the eccentricity of the orbit is not zero anymore, the attractor is periodic with period one, which means that the spacecraft crosses the Poincaré section with the single set of coordinates shown in Table 3.

**Table 3:** Coordinates of the attractors  in the phase-space of the Poincaré section when considering *J*_2_ in polar orbits for a thrust proportional to the radius of the orbit.

	values for the parameter *α*_1_ (10^−4^(*uT*)^−2^)			
	*α*_1_ = 1.2	*α*_1_ = 1.5	*α*_1_ = 2.0	*α*_1_ = 2.5
*x*(*m*)	7015945.35215650	6991320.89415925	6985126.18986852	6983241.55544237
*y*(*m*)	|*y*| < 10^−9^	|*y*| < 10^−9^	|*y*| < 10^−9^	|*y*| < 10^−9^
$\dot{x}(m/s)$	0.00037892	0.00041423	0.00047159	0.00052294
$\dot{y}(m/s)$	7539.156133457	7552.43334311	7555.78451696	7556.80494032

**Figure 5:**
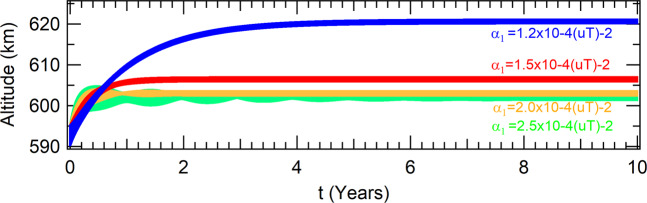
Effects of the perturbations due to *SRP* for a thrust proportional to the radius in the final altitude of the orbit of the spacecraft.

In the case where the solar radiation pressure is taken into account, the frame of reference is changed to one that rotates with the same angular velocity of the Sun around the Earth. The perturbation effect over the altitude is shown in Fig. 5. Similarly to the perturbation due to the *J*_2_ term for polar orbits, the attractors are moved to higher altitudes. The physical explanation is also easy to be made. The Solar Radiation Pressure acts opposite to the gravity of the Earth in the reference frame used here, when the spacecraft is in the semi-plane *x* > 0. So, its effect is equivalent to a reduction of the mass of the Earth, which gives higher final altitudes for the spacecraft. It has two opposite effects in the semi-plane *x* < 0, and the altitude decreases. It generates an oscilattion of the final altitude, as visible in Fig. 5. It means that a solar sail can be used to give different values for the final mean altitude and its range of oscilation of the spacecraft, acting as a passive control The attractor is also periodic with period one in the phase-space of the Poincaré section using the rotating frame of reference, and its coordinates are shown in Table 4.

**Table 4:** Coordinates of the attractors in the phase-space of the Poincaré section due to SRP for a thrust proportional to the radius.

	values for the parameter *α*_1_ (10^−4^(*uT*)^−2^)			
	*α*_1_ = 1.2	*α*_1_ = 1.5	*α*_1_ = 2.0	*α*_1_ = 2.5
*x*(*m*)	6998357.61992621	6984120.22756220	6980577.78678919	6979473.21206146
*y*(*m*)	|*y*| < 10^−9^	|*y*| < 10^−9^	|*y*| < 10^−9^	|*y*| < 10^−9^
$\dot{x}(m/s)$	−0.32090338	−0.29640297	−0.20906448	−0.05888977
$\dot{y}(m/s)$	7545.73024554	7553.45365269	7555.42601747	7556.05885375

**Figure 6:**
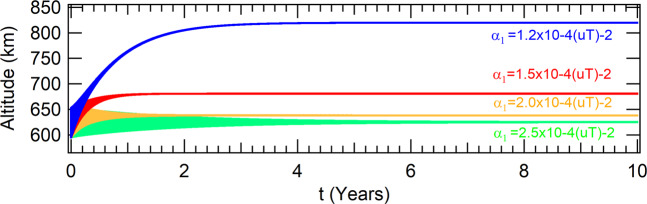
Effects of the perturbations due to the Moon for a thrust proportional to the radius in the final altitude of the orbit of the spacecraft.

Another perturbation to be considered is the gravitational potential of the Moon. Once again, to keep the dimension of the phase-space of the Poincaré section unchanged, the evaluations were done in a frame of reference that rotates with the same angular velocity of the Moon around the Earth. In this case, the effect of the perturbation due to the gravitational influence of the Moon over the altitude of the spacecraft is shown in Fig. 6. The coordinates of the attractors in the rotating frame of reference is shown in Table 5.

**Table 5:** Coordinates of the attractors in the phase-space of the Poincaré section due to the Moon for a thrust proportional to the radius.

	values for the parameter *α*_1_ (10^−4^(*uT*)^−2^)			
	*α*_1_ = 1.2	*α*_1_ = 1.5	*α*_1_ = 2.0	*α*_1_ = 2.5
*x*(*m*)	7197877.23607033	7058760.78277839	7016200.18755208	7002968.32880616
*y*(*m*)	|*y*| < 10^−9^	|*y*| < 10^−9^	|*y*| < 10^−9^	|*y*| < 10^−9^
$\dot{x}(m/s)$	−0.00117907	−0.00093344	−0.00052700	−0.00004123
$\dot{y}(m/s)$	7422.96825977	7496.30004086	7519.167244835	7526.31877766

#### Brief conclusions of the perturbed system

The parameter *α*_1_ plays an important role in the altitude of the attractor: the higher it is, the stronger the contribution of the term *α*_1_(*r*_0_ − *r*) of Eq. 4 to the thrust, and the closer the attractor is to *r*_0_. The displacements of the attractors are in the direction of higher altitudes for all kinds of perturbations shown here, except in the case of equatorial orbits due to the inclusion of the *J*_2_ term of the gravity field of the Earth, because it increases the effective mass of the central body. The gravitational effect of the Moon has the highest influence in the net displacement from *r*_0_, in comparison with other perturbations. The last important result is that, for all perturbed cases studied in this work, the spacecraft always evolved to periodic attractors with period one in their respective frame of reference. This can be helpful to track it, since it will always cross its respectives coordinates from time to time.

### Thrust proportional to the magnitude of the velocity and inversely proportional to the radius

In this subsection, we investigate a thrust used to maintain the spacecraft in attractors at desired altitudes that is proportional to the angular velocity *v*/*r*. When the spacecraft if falling into the Earth, its velocity is increased and its radius is decreased, hence the magnitude of the thrust is increased and the result is that it tends to evolve to its respective attractor. In this case, the magnitude of the thrust *τ* is given by

5 $$\tau ={\alpha }_{2}\frac{v}{r}.$$

**Figure 7:**
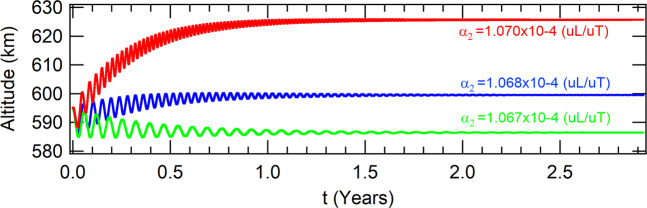
The altitudes of the different attractors are shown in red, blue and green, for the parameters *α*_2_ equals to *α*_2_ = 1.070 × 10^−4^(*uL*/*uT*), *α*_2_ = 1.068 × 10^−4^(*uL*/*uT*) and *α*_2_ = 1.067 × 10^−4^(*uL*/*uT*), respectively. The other parameters are *β* = 1000 × *β*_*ISS*_ and *a*_0_ = *r*_0_ − 5 *km*.

The spacecraft evolves to the attractors shown in Table 6 for three different values of the parameter *α*_2_. The evolutions of the respective altitudes are shown in Fig. 7. The higher this parameter, the higher the altitude of the attractor.

**Table 6:** Coordinates of the attractors in the phase-space of the Poincaré section due to *J*_2_ in equatorial orbit for thrust proportional to the radius.

	values for the parameter *α*_2_ (10^−4^(*uL*/*uT*)		
	*α*_2_ = 1.067	*α*_2_ = 1.068	*α*_2_ = 1.070
*x*(*m*)	6964560.37846288	6977620.96701989	7003778.84836227
*y*(*m*)	|*y*| < 10^−9^	|*y*| < 10^−9^	|*y*| < 10^−9^
$\dot{x}(m/s)$	$|\dot{x}| < {10}^{-8}$	$|\dot{x}| < {10}^{-8}$	$|\dot{x}| < {10}^{-8}$
$\dot{y}(m/s)$	7565.22821813	7558.14467111	7544.01729790

#### Linear stability of the unperturbed system

Once again, the orbits of the spacecraft at the attractor are circular for all the three attractors shown in Fig. 7. Thus, they can be considered fixed points in appropriate rotating frames of references. By solving the characteristic equation of the respective linearized system, it was found that all these attractors are linearly unstable.

#### Perturbed case

**Figure 8:**
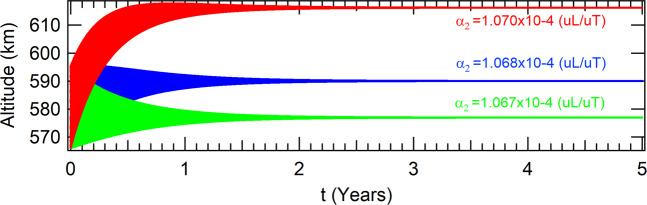
Perturbations due to *J*_2_ in equatorial orbits for a thrust proportional to the velocity and inversely proportiuonal to the radius of the orbit of the spacecraft.

**Figure 9:**
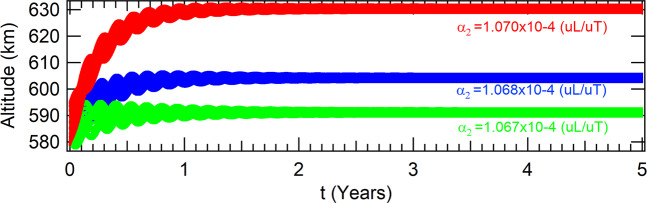
Perturbations due to *J*_2_ in polar orbits for a thrust proportional to the velocity and inversely to the radius of the orbit of the spacecraft.

In the case where the perturbation due to the *J*_2_ term of the gravitational potential of the Earth is taken into account for an equatorial orbit, the spacecraft is found in the attractor which coordinates in the Poincaré section is given in Table 7, for the same values of the parameter *α*_2_ evaluated in the non perturbed case. The respective altitudes are shown in Fig. 8. In the case of a polar orbit, the coordinates of the attractors are shown in Table 8 and the respective altitudes are shown in Fig. 9.

**Table 7:** Coordinates of the attractors in the phase-space of the Poincaré section to *J*_2_ in equatorial orbit for a thrust proportional to the velocity and inversely proportional to the radius of the orbit of the spacecraft.

	values for the parameter *α*_2_ (10^−4^(*uL*/*uT*))		
	*α*_2_ = 1.067	*α*_2_ = 1.068	*α*_2_ = 1.070
*x*(*m*)	6955061.54361013	6968139.95931161	6994333.34424211
*y*(*m*)	|*y*| < 10^−9^	|*y*| < 10^−9^	|*y*| < 10^−9^
$\dot{x}(m/s)$	$|\dot{x}| < {10}^{-8}$	$|\dot{x}| < {10}^{-8}$	$|\dot{x}| < {10}^{-8}$
$\dot{y}(m/s)$	7575.56022172	7568.42828979	7554.20496085

**Table 8:** Coordinates of the attractors in the phase-space of the Poincaré section considering *J*_2_ for polar orbits and a thrust proportional to the velocity and inversely proportional to the radius of the orbit of the spacecraft.

	values for the parameter *α*_2_ (10^−4^(*uL*/*uT*))		
	*α*_2_ = 1.067	*α*_2_ = 1.068	*α*_2_ = 1.070
*x*(*m*)	6970883.44423619	6983932.19757493	7010066.50793921
*y*(*m*)	|*y*| < 10^−9^	|*y*| < 10^−9^	|*y*| < 10^−9^
$\dot{x}(m/s)$	0.00012198	0.00012164	0.000120963
$\dot{y}(m/s)$	7563.50643237	7556.43094902	7542.31956748

**Figure 10:**
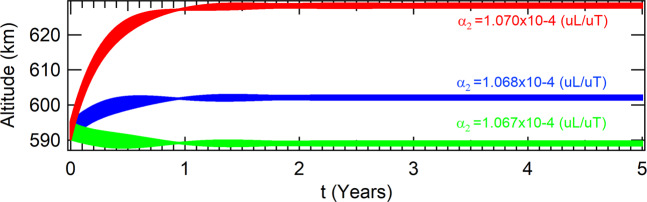
Perturbations due to *SRP* for a thrust proportional to the velocity and inversely proportional to the radius of the orbit of the spacecraft.

In the case where the solar radiation pressure is taken into account, the spacecraft will evolve to the attractors shown in Table 9, which altitudes evolutions in time can be seen in Fig. 10.

**Table 9:** Coordinates of the attractors in the phase-space of the Poincaré section including SRP for a thrust proportional to the velocity and inversely proportional to the radius of the orbit of the spacecraft.

	values for the parameter *α*_2_ (10^−4^(*uL*/*uT*))		
	*α*_2_ = 1.067	*α*_2_ = 1.068	*α*_2_ = 1.070
*x*(*m*)	6966592.41369304	6979662.15516907	7005838.44220465
*y*(*m*)	|*y*| < 10^−9^	|*y*| < 10^−9^	|*y*| < 10^−9^
$\dot{x}(m/s)$	−0.174013958	−0.174290219	−0.1748698966
$\dot{y}(m/s)$	7563.02151982	7555.93425592	7541.79943999

**Figure 11:**
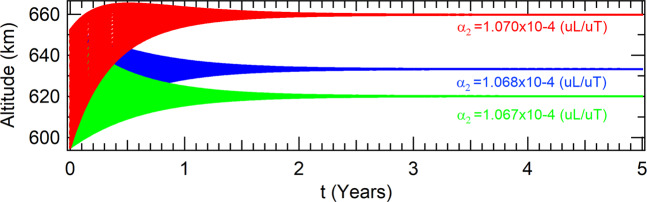
Perturbations due to the Moon for a thrust proportional to the velocity and inversely proportional to the radius.

For the perturbation due to the gravitational attraction of the Moon, the spacecraft evolves to the attractors shown in Table 10. Its altitudes as function of time are shown in Fig. 11.

**Table 10:** Coordinates of the attractors in the phase-space of the Poincaré section including Moon for a thrust proportional to the velocity and inversely proportional to the radius of the orbit of the spacecraft.

	values for the parameter *α*_2_ (10^−4^(*uL*/*uT*))		
	*α*_2_ = 1.067	*α*_2_ = 1.068	*α*_2_ = 1.070
*x*(*m*)	6998127.97657272	7011346.92909045	7037823.34032799
*y*(*m*)	|*y*| < 10^−9^	|*y*| < 10^−9^	|*y*| < 10^−9^
$\dot{x}(m/s)$	−0.00043678	−0.00043801	−0.00044046
$\dot{y}(m/s)$	7528.93992007	7521.78798627	7507.52364247

### A more general model for the thrust

In this case, the thrust over the spacecraft is given by

6 $$\tau ={\alpha }_{2}\frac{v}{r}+{\alpha }_{1}({r}_{0}-r)+{\alpha }_{0}\beta {v}_{0}^{2}.$$

**Figure 12:**
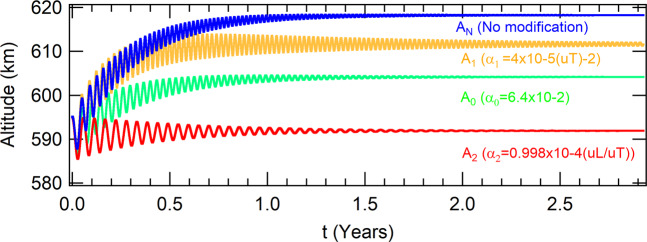
The evolution of the spacecraft to the attractors *A*_*N*_, *A*_0_, *A*_1_ and *A*_2_ as functions of time. The parameters of the attractor *A*_*N*_ are *α*_0_ = 6.5 × 10^−2^, *α*_1_ = 1 × 10^−5^(*uT*^−2^) and *α*_2_ = 1 × 10^−4^(*uL*/*uT*). The parameters of the attractors *A*_0_, *A*_1_ and *A*_2_ are the same of *A*_*N*_, except *α*_0_, *α*_1_ and *α*_2_, respectively.

The parameter *r*_0_ is set to *r*_0_ = *r*_*e*_ + 600 *km* and the initial condition is 5 *km* below this value (*a*_0_ = *r*_0_ − 5 *km*). The other parameters are fixed in the values *α*_0_ = 6.5 × 10^−2^, *α*_1_ = 1 × 10^−5^(*uT*^−2^) and *α*_2_ = 1 × 10^−4^(*uL*/*uT*) and they are modified one at a time in each color in Fig. 12. The attractors are shown in Table 11.

**Table 11:** Coordinates of the attractors in the phase-space of the Poincaré section.

	Coordinates as functions of the parameters			
	no modification	*α*_0_ = 6.4 × 10^−2^	*α*_1_ = 4 × 10^−5^(*uT*^−2^)	*α*_2_ = 9.998 × 10^−5^(*uL*/*uT*)
*x*(*m*)	6996351.67397836	6982273.22670731	6989755.98126234	6970012.75529358
*y*(*m*)	|*y*| < 10^−9^	|*y*| < 10^−9^	|*y*| < 10^−9^	|*y*| < 10^−9^
$\dot{x}(m/s)$	$|\dot{x}| < {10}^{-8}$	$|\dot{x}| < {10}^{-8}$	$|\dot{x}| < {10}^{-8}$	$|\dot{x}| < {10}^{-8}$
$\dot{y}(m/s)$	7548.02051789	7555.62627132	7551.58092009	7562.26864358

#### Linear stability of the unperturbed system

The orbits of the spacecraft at the four attractors shown in Fig. 12 can also be considered as circular ones. By solving the characteristic equation of the respective linearized system in appropriate frames of references, it was found that the attractor *A*_*N*_, *A*_1_ and *A*_2_ are stable, while the attractor *A*_0_ is unstable.

#### Perturbed case

**Figure 13:**
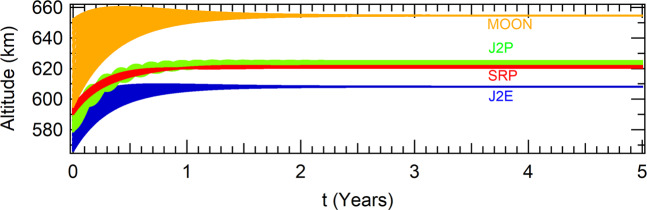
The influence of several types of perturbations over the attractor *A*_*N*_ shown in Fig. 12, which parameters are *α*_0_ = 6.5 × 10^−2^, *α*_1_ = 1 × 10^−5^(*uT*^−2^) and *α*_2_ = 1 × 10^−4^(*uL*/*uT*).

Considering the values for the parameters of the “no modification” blue curve of Fig. 12, in the case where the perturbations due to the oblateness of the Earth is considered for polar and equatorial orbits, the SRP and the gravitation effect of the Moon, the spacecraft evolves to the attractors shown in Table 12. The altitudes as functions of time are shown in Fig. 13.

**Table 12:** Perturbations with a more general model to thrust.

	Coordinates as functions of the perturbations			
	*J*_2_ Eq.	*J*_2_ Polar	SRP	Moon
*x*(*m*)	6986211.37428672	7002984.55909160	6998600.80209396	7032733.18747531
*y*(*m*)	|*y*| < 10^−9^	|*y*| < 10^−9^	|*y*| < 10^−9^	|*y*| < 10^−9^
$\dot{x}(m/s)$	$|\dot{x}| < {10}^{-8}$	0.00014044	−0.18507844	−0.00047047
$\dot{y}(m/s)$	7558.60669434	7546.13569400	7545.6963499	7510.259769

The higher the parameter *α*_0_, the higher the altitude of the attractor (and vice-versa), but this parameter is also related to the transient time. The lower values of *α*_0_, the higher the time to reach the attractor. The higher the parameter *α*_1_, the closer the attractor is to *r* = *r*_0_, but, also, the higher its value, the higher is the amplitude of oscillations, and, as a consequence, more time is required for the system to reach the attractor. The higher the parameter *α*_2_, the higher the altitude of the attractor. It is also lower the time that the system requires to reach it. These parameters are important for a mission designer. As an example, in the case where a spacecraft needs to reach a previous determined altitude in the lower possible time, the parameter *α*_1_ should be reduced.

### Transfers among attractors

In this section, the transfers among attractors will be studied using a basin of attraction. In the case where a spacecraft is bounded in an attractor and that it should be transferred to another altitude, the evaluation of the basin of attraction of the final attractor can show if the transfer can be done by a change of parameters.

There are several applications of this idea, like to make a transfer orbit to place the spacecraft in a different altitude to make observations from another location, rendezvouz with another spacecraft, using an altitude that requires less fuel consumption for maintenance, to make the spacecraft to reenter the atmosphere to be destroyed; or even to escape from the Earth. As an example, we suppose that a spacecraft should be transferred to an altitude *alt* = 618216 *m* through the attractor described as “No modification” shown in Fig. 12 and Table 11. The parameters of the thrust must be fixed in the values *α*_0_ = 6.5 × 10^−2^, *α*_1_ = 1 × 10^−5^(*uT*^−2^) and *α*_2_ = 1 × 10^−4^(*uL*/*uT*), but the transfer will succeed only if the coordinates of the spacecraft in its phase-space are within the basin of attraction of the final attractor.

**Figure 14:**
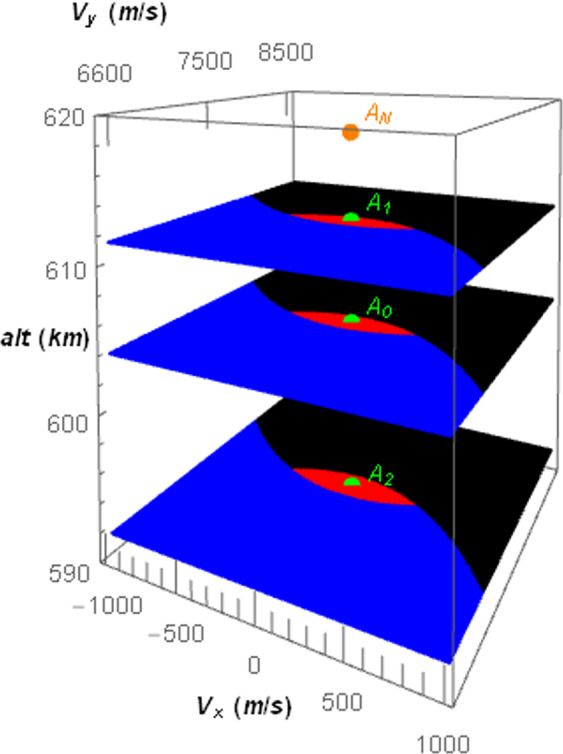
Planar sections of the basin of attraction (in red) of the attractor shown in orange for the parameters *α*_0_ = 6.5 × 10^−2^, *α*_1_ = 1 × 10^−5^(*uT*^−2^) and *α*_2_ = 1 × 10^−4^(*uL*/*uT*) for several values of the initial altitude. The attractors for a modified parameter - *A*_0_ for *α*_0_ = 6.4 × 10^−2^, *A*_1_ for *α*_1_ = 4 × 10^−5^(*uT*^−2^) and *A*_2_ for *α*_2_ = 9.998 × 10^−5^(*uL*/*uT*) are shown in green. The coordinates of these attractors in the phase-space of the Poincaré section are also shown in Table 11.

**Figure 15:**
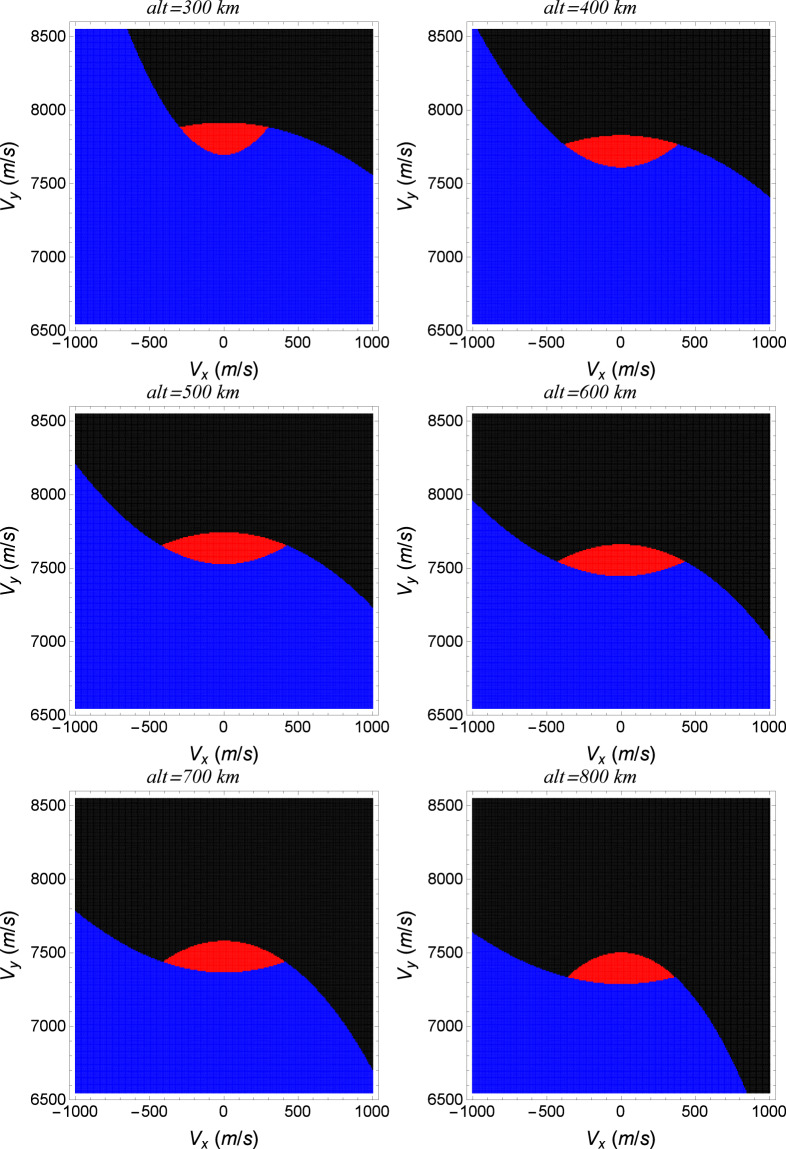
Basins of attraction of the attractor shown in Table 11 are shown in red for the parameters *α*_0_ = 6.5 × 10^−2^, *α*_1_ = 1 × 10^−5^(*uT*^−2^) and *α*_2_ = 1 × 10^−4^(*uL*/*uT*) for several values of the initial altitude.

In Fig. 14, several planar sections of the basin of attraction with respect to the “No modification” attractor are shown in the phase-space of the Poincaré section. The regions in red will evolve to the attractor, while the initial conditions in blue will make the spacecraft to fall in the Earth and the initial conditions in black will make the spacecraft to escape from the Earth. Numerically, the conditions in red will reach the attractor with a precision of 10^−3^ in position and velocities coordinates in the IS, while the conditions in blue will evolve the spacecraft to fall to altitudes lower than 200 *km*, (that will end in reentry) in the atmosphere of the Earth, and the conditions in black reach altitudes higher than 1000 *km*. Note that only the initial conditions of the spacecraft in red can evolve it to the “No modification” attractor. The coordinates in the phase-space of the attractors shown in Fig. 12 and Table 11 for several values of modified parameters are also shown in Fig. 14, which are placed in altitudes equal to 592, 604 and 612 *km*. Note that these attractors are contained in the red region of the plane, which means that the spacecraft can be transferred from one of these attractors to the “No modification” attractor by a change of parameters of the thrust. In Fig. 15, planes of the basin of attraction are shown for several values of altitude. They represent the conditions (components of the velocity) in which a spacecraft can be set such that it will evolve to the “No modification” attractor as function of the respective altitudes.

## Conclusions

In this work, it is shown how a spacecraft equipped with a thrust can be bounded in specific altitudes in LEO, taken into consideration the drag, the gravitational influence of the Earth and the Moon, the SRP, and the oblateness of the Earth for equatorial and polar orbits for several types of thrusts. In the cases where only the Earth’s gravity, the drag and the thrust are taken into consideration, the orbit is circular for all the cases investigated here.

Some attractors correspond to circular orbits and they become fixed points in proper rotating frames of reference. In the case where the model to the thrust is given by Eq. (4), these fixed points are linearly stable for all the attractors shown in section 3.1 when no perturbation is taken into consideration. The attractors to where the spacecraft is evolved are unstable for the cases evaluated with the second model of the thrust shown in section 3.2 in the case where there is no perturbation over the system. The third model shown in section 3.3 contains terms that come from the first and the second model, hence, its linear stability depends on the magnitude of each term. Thus, the linear stability analyzes indicated that the first model to the thrust shown in section 3.1 is the most reliable among the three models shown in this paper.

All types of perturbations change the attractor to higher altitudes with non circular orbits, except for the case where the perturbation is given by the *J*_2_ term in equatorial orbits. On the other side, in appropriates frames of reference, the orbit is still periodic with a single period in the phase-space of the Poincaré section, which is an important result that can be used to simplify the prediction of the motion of a spacecraft. It facilitates its tracking over the time, which can be used to predict its motion around the Earth. Note that the drag is used to help the spacecraft to evolve to attractors, therefore, their basin of attraction are also important to predict the evolution of such a spacecraft. In this sense, transfers among different attractors are also shown and investigated in the present paper, performed by a change of the parameters of the thrust. We shown that the basin of attraction of an attractor can be used to investigate the boundaries in the phase-space that the spacecraft is placed in order to transfer it from a wide range of altitudes and velocities to final altitude and velocity desired for a mission.

In astrodynamics, in the perturbation theory, each perturbation is analyzed separately to observe and to quantify the main effects of the specific perturbation along the orbit. Under the same assumption, we analyzed the influence of the drag with multiple configurations of other perturbations and initial conditions, only to see the evolution of the orbit and the time to stabilize into the attractor. The sensitivity showed to be a function of the initial altitude and the control coefficients presented, but was not too small and the results are relevant.

The continuous low thrust was applied with a constant thrust force along the time, with maximum duration of ten years. The new advances in electric propulsion allow the implementation of propulsion systems with specific impulse larger than 10.000 *s*. It is expected that new advances in this technology increase the specific impulse and the thrust duration.

Of course that a limited set of parameters and perturbations is used in this work, but the method can be extended to adapt its values to specific missions, in which intrinsic perturbations must be taken into account. Additionally, only two dimensional trajectories were investigated in this work. Of course that three dimensional orbits taken into account all the perturbation together would represent a more realistic and complete system, but it is left for further researches. Note that we used specific frames of reference according to each kind of perturbations in order to turn the equations of motion explicitly independent of time. In the case where all the perturbations are taken into account, the phase-space would have one more dimension, due to this time dependence of the equations of motion plus two dimensions due to the space span in one more degree of freedom. In this case, the analyses would be much harder to interpret in the seven dimensions of the phase-space, and we also leave this task to a future work.
